# Match Running Performance in UEFA Champions League: Is There a Worthwhile Association with Team Achievement?

**DOI:** 10.3390/biology11060867

**Published:** 2022-06-06

**Authors:** Toni Modric, Sime Versic, Paweł Chmura, Marek Konefał, Marcin Andrzejewski, Igor Jukic, Patrik Drid, Suncica Pocek, Damir Sekulic

**Affiliations:** 1 Faculty of Kinesiology, University of Split, 21000 Split, Croatia; dado@kifst.hr; 2 HNK Hajduk Split, 21000 Split, Croatia; 3 Department of Team Games, Wrocław University of Health and Sport Sciences, 51612 Wrocław, Poland; pawel.chmura@awf.wroc.pl; 4 Department of Human Motor Skills, Wrocław University of Health and Sport Sciences, 51612 Wrocław, Poland; marek.konefal@awf.wroc.pl; 5 Department of Methodology of Recreation, Poznań University of Physical Education, 61871 Poznań, Poland; andrzejewski@awf.poznan.pl; 6 Faculty of Kinesiology, University of Zagreb, 10000 Zagreb, Croatia; igor.jukic@kif.hr; 7 Faculty of Sport and Physical Education, University of Novi Sad, 21 000 Novi Sad, Serbia; patrikdrid@gmail.com (P.D.); suncicapocekfsfv@gmail.com (S.P.)

**Keywords:** football, physical demands, playing positions, success, elite players

## Abstract

**Simple Summary:**

This study demonstrated that running performance (RP) is not the factor which defines team achievement in UEFA Champions League (UCL) group stage, indicating that the success of the teams who compete at the elite-level soccer is not influenced by pure physical performance. Although RP will not assure success, considering high physical demands observed in UCL, conditioning of the players who compete at the elite-level soccer should be at the highest possible level. These findings could help soccer coaches to optimize the training process of elite soccer players.

**Abstract:**

Although running performance (RP) is considered an important factor of success in soccer, there is a lack of studies to examine this issue in highest-level soccer competition, such as UEFA Champions League (UCL). Therefore, the main objective of this study was to analyse players’ RP according to the achievement of their teams in UCL. In addition, position specific RP of the players who competed in the UCL was evaluated. The players’ RPs (*n* = 244) were collected during UCL group stage matches (*n* = 20) in the 2020/21 season using semiautomatic optical system InStat Fitness. A team’s achievement was defined by qualification of the team from the group stage into the knockout stage of the UCL, and by total group points earned at the end of the UCL group stage. Linear mixed models and Pearson’s correlation were used to examine differences in players’ RP according to the achievement of their teams. Results indicated (i) similar values of RP irrespective of whether the teams qualified from the group stage into the knockout stage of the UCL, and (ii) trivial-to-small correlations between RP and total group points. Such findings show that players’ RP was poorly related to the achievement of their teams in the UCL group stage, indicating trivial influence of RP on success in elite-level soccer.

## 1. Introduction

Interest in match analysis of soccer with the primary objectives being to record and analyse the physical demands of the players has increased over the last three decades [1,2,3,4,5,6,7,8]. Today, it is well known that soccer players can cover between 9 and 14 km during the matches, performing 5–15% of that distance in high intensity running [9,10]. These demands vary according to the different playing positions of the players in the match [11]. Specifically, central midfielders cover the greatest overall distance, while external players (i.e., wide midfielders and fullbacks) cover the greatest distance in terms of high-intensity running [12].

However, a large body of published research indicates that physical demands in soccer may vary on many multiple factors [13,14,15]. For example, tactical roles [16], physical capacities [17], and contextual factors such as the match status (win, draw, lose), match location (home, away), or and opponents’ level (top, middle, bottom) have all been demonstrated to affect running performance (RP) in soccer matches [18,19]. Such large interests in match RP ultimately forcibly shaped contemporary opinions, with researchers and practitioners frequently emphasizing the importance of RP in elite soccer [20].

The possible explanation for these beliefs is the fact that key match activities, which are crucial for final match result, may be affected by greater RP, especially at higher speeds [21,22,23]. For example, to successfully attack the last third of the pitch (i.e., by forwards), to perform efficient counterattacks (i.e., by wide midfielder), to go in the last third of the pitch for crossing (i.e., by fullbacks) or to perform defensive transition (i.e., by central midfielders and central defenders), players should create spatio-temporal advantages over the opponent players. As this can be done only by running at higher speeds, a higher amount of such activities actually provokes greater high-speed running and sprinting. In view of this, greater RP at higher speed may be associated with successful team performance. However, previous studies which analysed the RP of successful and unsuccessful teams from elite soccer competitions reported inconsistent findings [21,24,25,26].

Briefly, Di Salvo et al. reported that English Premier League players from less successful teams covered a greater high-intensity distance (above 19.8 km/h and 25.2 km/h) than better teams [21]. Rampinini et al. compared the RP of the first five and last five teams in Italian Seria A and demonstrated that players on the lower-ranked teams accumulated a greater total- and high-intensity distance covered (19+ km/h) than teams who were ranked more highly [24]. In contrast, Chmura et al. and Andrzejewski et al. in their studies reported that players on specific playing positions (e.g., wide midfielders and forwards) ran significantly greater distances at higher speeds when their teams achieved success in a single match [23,27]. On the other hand, Hoppe et al. reported that match RP was not significantly correlated with the final points accumulated among German Bundesliga soccer teams [25], which is supportive to findings of Clemente et al., who recently revealed that both successful and unsuccessful teams from Spanish La Liga presented the same RPs at higher velocities (above 14 km/, 21 km/h, and 24 km/h) [26].

Considering that the authors of these studies drew their conclusions observing teams that belong to only one country, it is possible that these inconsistencies might be due to geographical, cultural, historical, and social aspects of the observed competition [28,29,30]. In addition, it possible that the evolving nature of soccer had a large effect on results from aforementioned studies [31]. Irrespective of causality, it is still unclear whether RP is important for achieving success in elite soccer. To clarify this issue, analysis of soccer competition at the elite level which includes teams from different countries may be more appropriate for drawing conclusions. Undoubtedly, the most elite and most prestigious soccer clubs’ competition that includes teams from different countries is UEFA Champions League (UCL) [32].

To the best of our knowledge, RP of successful and unsuccessful teams in UCL was examined only once. Specifically, authors of the current study evaluated associations between team achievement and RP in offensive and defensive phases of the game in their previously published study [33]. As it has been found that RP in both offensive and defensive phases of game may affect team achievement in UCL, we believe it is worthwhile to analyse association between overall RP and success in detail. Moreover, it has previously been suggested that more studies, based on a more complex statistical approach, across various European top leagues are needed to clarify association between RP and success in soccer [25].

Aside from our previous study, it is important to emphasize that RP in UCL in general was not sufficiently investigated. To date, only few studies analysed RP of soccer players that competed in UCL [1,34,35]. Briefly, Di Salvo et al. and Minano-Espin et al. analysed only RP at higher speeds, while Bradley et al. focus was on gender differences in RP [1,34,35]. For all these reasons, providing new evidence that may enable a detailed understanding of RP in UCL and clarify whether RP is important for achieving success in elite soccer seems reasonable. Therefore, the main objective of this study was to analyse players’ RP according to the achievement of their teams in UCL. In addition, position specific RP of the players who competed in the UCL was evaluated.

## 2. Materials and Methods

### 2.1. Participants and Design

RPs of elite soccer players (*n* = 179) from teams (*n* = 24) that competed in the group stage of UCL during the 2020–21 season were included as cases in this study. All RP data were obtained from the 20 matches from groups A (*n* = 3), B (*n* = 3), C (*n* = 4), E (*n* = 4), F (*n* = 3), and G (*n* = 3). As suggested previously, only the RPs of outfield players (i.e., goalkeepers were not included in analysis) who played in whole matches were analysed [1,34,35]. As a result, 244 match RPs were retrieved and used as cases for this study.

Players’ RPs were classified by match playing position as: central defenders (CD; *n* = 79), fullbacks (FB; *n* = 65), central midfielders (CM; *n* = 55), wide midfielders (WM; *n* = 28) and forward (FW; *n* = 17) [1,34]. Later, players’ position-specific RPs were classified into two subgroups according to the achievement of their teams. The first group (*n* = 99) included the RPs of the players from teams that finished in the first or second position in the groups, whereas the second group (*n* = 145) included the RPs of the players from teams that finished in the third or fourth position after all the matches were played in the group stage. 

All data were anonymised in accordance with the declaration of Helsinki to ensure player and team confidentiality. This investigation was part of a previously published paper with a different approach to the same subjects’ data [33]. Both investigations were approved by the ethical board of Faculty of Kinesiology, University of Split. 

### 2.2. Procedures

The RP data were collected using an optical semiautomatic system InStat Fitness (Instat Limited, Limerick, Republic of Ireland) with a sampling frequency of 25 Hz. The reliability of the system has been demonstrated by comparison of data to the Vicon motion system (Oxford Metrics Ltd., Oxford, UK) at five velocity bands (0–7 km/h, 7–15 km/h, 15–20 km/h, 20–25 km/h, and 25+ km/h). This procedure included analysis of mean velocity difference compared to Vicon (m/s) and mean position difference (m) compared to Vicon, and the system has passed the official Fédération Internationale de Soccer Association (FIFA) test protocol for Electronic and Performance Tracking Systems (EPTS) (authorization number: 1007382; report available on the official FIFA webpage: https://www.fifa.com/technical/football-technology/resource-hub?id=aca57303eb0449f2835ac891b1beeb24; accessed on 30 May 2022). Finally, previous research showed that this system is very accurate with high levels of absolute and relative reliability [35].

The RP variables included total distance covered (m) and distance covered in five speed categories: low-intensity (<14.3 km/h), running (14.4–19.7 km/h), high-speed running (19.8–25.1 km/h), sprinting (>25.2 km/h) and high-intensity running (>19.8 km/h). 

Team achievement in this study was defined by two criteria: (i) qualification of the team from the group stage into the knockout stage of the UCL (qualification) and (ii) total group points earned at the end of the group phase of the UCL competition (points). The UCL group contained 8 groups, and each group consisted of 4 clubs. After 6 matches were played in the group, the first- and second-ranked teams from each group advanced for competition in the knockout stage. Therefore, teams were classified as “qualified” (placed 1st and 2nd in the group phase) and “nonqualified” (placed 3rd and 4th in the group phase), which was observed as the first criterion of team achievement. Additionally, the total group points after all matches played in the group stage of the UCL were used as secondary criteria of team achievement. Data are presented as means ± standard deviations.

### 2.3. Statistics

The normality of the distributions was tested using the Kolmogorov–Smirnov test procedure, while Levene’s test was used to check homoscedasticity. Data are presented as the means and standard deviations. 

Due to the hierarchical structure of data whereby units of analysis (individual match observations) are clustered in higher units (players), who are again clustered by higher units (teams), linear mixed models were constructed to examine the effect of “playing position” and “qualification” on RP, considering the player and team identity (random effects) [36,37,38]. The assumptions of homogeneity and normal distributions of residuals were verified for each model, without revealing specific problems. Pair-wise comparisons were conducted via Bonferroni-adjusted post hoc tests. The effect size (ES) was evidenced throughout the calculation of partial eta squared (η^2^) (>0.02 is small; >0.13 is medium; >0.26 is large ES) [39]. 

Pearson’s correlation was used to identify associations between RP and total group points. Correlations were calculated for the total sample and stratified by playing position, with the r coefficient classification as previously suggested: r ≤ 0.35 indicates a low or weak correlation, r = 0.36 to 0.67 indicates a modest or moderate correlation, r = 0.68 to 1.0 indicates a strong or high correlation, and r > 0.90 indicates a very high correlation [40]. Due to the multiple correlations, a Bonferroni method for family-wise error rate correction was used, and an adjusted significance level of *p* ≤ 0.001 was applied [41]. 

For all analyses, SPSS software Version 25 (IBM, Armonk, NY, USA) was used, and *p* < 0.05 was considered statistically significant.

## 3. Results

Significant differences among playing positions were evidenced for all RP variables, with large ES found for total distance covered, running zone distance covered, high-speed running, and high-intensity running. Additionally, medium ES were found for low-intensity running and sprinting. In detail, CM covered the longest total distance, low-intensity running distance and distance while running (all significant post-hoc differences when compared to all other playing positions). FB and WM achieved the greatest amount of sprinting distance (both significant post-hoc differences when compared to CD and CM). High speed running and high-intensity running were lowest among CD (both significant post-hoc differences when compared to all other playing positions) and greatest among WM (Table 1).

**Figure 1:**
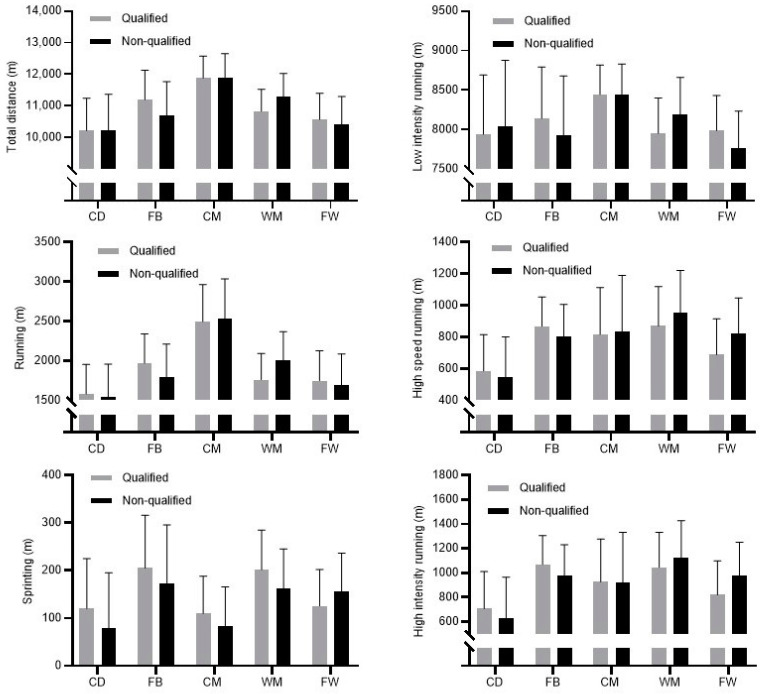
Descriptive statistics and differences in running performances for players at different playing positions (CD—central defenders, FB—full backs, CM—central midfielders, WM—wide midfielders, FW—forwards) according to their team’s qualification from the group stage of the UCL.

Figure 1 presents descriptive parameters and differences in RP for each playing position based on successful/unsuccessful qualification of the teams from the group stage of the UCL. For all playing positions, no differences were evidenced in players’ RP irrespective of whether the teams qualified from the group stage into the knockout stage of the UCL or not.

In general, RPs were not related to total points earned when observed for the total sample or separately for each playing position. More precisely, of 36 correlations, only two reached statistical significance (*p* < 0.05), with running of WM being significantly negatively correlated with total earned points of the team (16% of the common variance) and sprinting of the whole team being positively correlated with earned points (<2% of the common variance). However, none of these correlations reached an adjusted level of significance (*p* < 0.001) (Table 2). 

## 4. Discussion

The main objectives of this study were to evaluate the position-specific RP of the players who competed in the UCL, and to analyse players’ RP according to the achievement of their teams in UCL. The findings show that the RPs of players were poorly related to the achievement of their teams in the UCL group stage, indicating trivial influence of RP on success in elite-level soccer. In addition, significant differences in RP among playing positions were evidenced (all moderate to large effect sizes), supporting previous conclusions that RP appears to be related to playing position due to the different main duties of players on different playing positions [1,42]. 

Specifically, CMs’ main duty in the game is connection between defense and attack, and such tactical roles require them to achieve greater distances [43,44]. Indeed, we evidenced that CMs covered significantly greatest total distance of all playing positions. In detail, during the UCL group stage, CM reached 11,879 m on average over the total distance. These results are in the line with reports from elite soccer competitions such as English Premier League, Spanish La Liga and Europa League (11,450 m, 12,027 m and 11,760 m, respectively) [9,43,45], while reports from lower-ranked competitions such as Croatian and Brazilian first divisions revealed CMs’ slightly lower values (11,155 m and 10,144 m, respectively) [10,42].

It is well known that players who play by the side of the pitch (i.e., FB and WM) in general cover the greatest amount of high-intensity running (i.e., high speed running + sprinting) due to the their frequent involvement into the attack [21,45]. Supporting this, our results indicated that WMs and FBs outperformed all other players in high-intensity running (i.e., both in high-speed running and sprinting). Specifically, the WMs and FBs in UCL covered 1098 m and 1017 m, respectively. These results are similar to the amount reported for elite soccer competitions [43,45], but approximately 20–25% greater than in the lower-ranked competitions [10,42].

In general, CDs’ main game duties are related to the reactions or accelerations [10], and therefore they achieve the lowest total and high-intensity distance of all players [45,46,47]. Not surprisingly, we evidenced significantly lowest total and high-intensity distance among CDs. In detail, during the UCL group stage, CDs achieved 10,200 m in total, and 638 m of high high-intensity running, which is similar to reports from elite soccer competitions [43,45]. In contrast, reports from the lower-ranked competitions for CDs evidenced 30–60% less of overall distance, and 70–130% less of high-intensity running [10,42]. 

FWs’ sprint distance covered during the match is a highly important determinant of their overall game performance [10]. Moreover, previous studies reported that FWs in general utilize a large amount of high-intensity running during the matches [9,21,48]. Our results show that FWs who competed in UCL achieved on average 900 m of high-intensity running. Similar high-intensity distances were evidenced among FWs from elite soccer competitions [43,45], while in sub-elite soccer competitions FWs covered 50–130% less of high-intensity running [10,42].

Although comparing absolute values of RP in UCL with values obtained from the previous studies is not the main objective of our study, it is worthwhile to mention evident differences in RPs among different competitions and leagues. Thus, irrespective of the fact that various systems of match analytics were used, which could partially influence the established differences between competitions to some extent [49], comparisons of our results with reports from elite soccer competitions suggest that UCL is one of the most physically demanding soccer competitions. However, although it is often postulated that a high level of match RP is important for greater achievement among professional soccer teams [25], this study does not support such considerations for UCL. 

Specifically, our results did not indicate a significant association between players’ RPs and the achievement of their teams. We found similar values of RPs irrespective of whether the teams qualified from the group stage into the knockout stage of the UCL. Additionally, trivial-to-small correlations between total group points and total distance covered and distance covered in different speed indicated a lack of association between RP and team achievement in the UCL group stage. Such results are in contrast with our previously published study in which significant associations between RP and team achievement were observed [33]. Briefly, it was demonstrated that successful teams played at a higher game pace in the offensive phase of game (i.e., when team has ball in possession) and achieved greater running efforts in the defensive phase of game (i.e., when opponent has ball in possession). Taking it into account along with previous studies which reported greater RPs of elite English and Italian players from less successful teams [21,24], results from current study may seem surprising.

However, such results (e.g., a nonsignificant association between RP and achievement) are supportive of some more recent considerations brought in other elite competitions [25,26]. Specifically, a very recent study revealed that the final position in the classification table of the Spanish La Liga did not depend on the team’s RP at higher speeds [26]. In addition, a study conducted among German Bundesliga players reported that match RP (i.e., total distance covered and distance covered at higher speed) was not significantly correlated with the final points accumulated in competition [25]. Therefore, it seems that players’ RP is poorly related to the achievement of their teams in the elite soccer. It is most likely that the overall technical and tactical effectiveness has a greater impact on results and a team’s final league ranking than RP, as previously suggested [21,26,33,50].

There were some potential limitations of the current. Specifically, due to the unavailability of RP data from all matches, we did not analyse all matches from the group stage of the UCL (i.e., we only noted 20 randomly selected matches). However, using a relatively small sample is a very common obstacle in studies involving players which compete at the highest level of soccer [33,48]. Furthermore, due to the methodological reasons, only those players completing entire matches (i.e., 90 min) were included for analysis, as suggested previously [1,51]. Finally, situational factors such as team and opposition quality or match location, which have been shown to influence RP in national soccer competitions [36,42,50], were not considered in the current study. However, differences in teams’ and opponents’ quality in UCL are most likely lower than in national competitions, and, consequently, influence on RP may be negligible. In addition, all observed matches were played without audience or with limited capacity in the stands due to the COVID-19 pandemic [52]; therefore, the influence of match location (i.e., home advantage) may be insignificant. 

## 5. Conclusions

The results from this study demonstrated that RP is not the factor which defines team achievement in UCL (i.e., defined by qualifying from the group stage and total points earned at the end of group stage), ultimately indicating that the success of the teams who compete in elite-level soccer is not influenced by pure physical performance. However, any conclusion regarding the eventual non-importance of the physical performance in soccer would be (at least) irresponsible. Namely, comparison with reports from elite competitions indicates that the UCL is one of the most physically demanding soccer competitions. Although RP will not assure success, players still must be able to handle high RP to participate in UCL. Therefore, it becomes clear that competing in the UCL requires the conditioning of the players to be at the highest possible level. These findings could help soccer coaches to optimize training process of elite soccer players. 

Considering that soccer is a highly complex sport incorporating interplay between physical and technical factors [53], future studies should analyse RP integrated with technical-tactical performance to gain a more holistic understanding of differences between more successful and less successful teams in the UCL. In addition, future studies should analyse RP more extensively, including (i) average calculated values of all players who played, (ii) accelerations and decelerations, (iii) values opened in the first and second half, (iv) values achieved in attack and defense and (v) values achieved in different periods of matches.

## Figures and Tables

**Table 1:** Descriptive statistics and differences in running performances among players at different playing positions (data are given as Mean ± SD in meters).

	CD	FB	CM	WM	FW	F	*p*	η2
TD	10,208 ± 907 ^†,‡,§^	10,937 ± 866 *^,‡^	11,875 ± 844 *^,†,§,||^	11,158 ± 75 *^,‡,||^	10,480 ± 727 ^‡,§^	42.54	(0.001)	0.50
LIR	7994 ± 587 ^‡^	8031 ± 563 ^‡^	8442 ± 549 *^,†,§,||^	8074 ± 496 ^‡^	7905 ± 468 ^‡^	10.23	(0.001)	0.22
R	1562 ± 418 ^†,‡,§^	1879 ± 401 *^,‡^	2510 ± 392 *^,†,§,||^	1953 ± 359 *^,‡^	1693 ± 348 ^‡^	57.47	(0.001)	0.56
HSR	557 ± 228 ^†,‡,§,||^	836 ± 219 *	835 ± 214 *	950 ± 195 *^,||^	756 ± 187 *^,§^	32.84	(0.001)	0.44
S	98 ± 103 ^†,§^	187 ± 100 *^,‡^	95 ± 97 ^†,§^	181 ± 90 *^,‡^	142 ± 88	12.88	(0.001)	0.24
HIR	655 ± 298 ^†,‡,§,||^	1025 ± 285 *	930 ± 277 *^,§^	1136 ± 249 *^,‡,||^	899 ± 239 *^,§^	29.92	(0.001)	0.42

Legend: TD—total distance; LIR—low-intensity running; R—running; HS—high speed running; S—sprinting, HIR—high-intensity running; * Significantly different (*p* < 0.05) from CD; ^†^ Significantly different (*p* < 0.05) from FB; ^‡^ Significantly different (*p* < 0.05) from CM, ^§^ Significantly different (*p* < 0.05) from WM; ^||^ Significantly different (*p* < 0.05) from FW; CD—central defenders, FB—full backs, CM—central midfielders, WM—wide midfielders, FW—forwards.

**Table 2:** Pearson’s correlations for players at different playing positions between running performances and total group points of theirs teams (results are given as Pearson’s coefficient (*p*)).

	CD	FB	CM	WM	FW	Total Sample
TD	−0.05	0.19	0.09	−0.37	0.24	0.04
	(0.64)	(0.13)	(0.54)	(0.06)	(0.36)	(0.57)
LIR	−0.21	0.12	−0.03	−0.28	0.27	−0.04
	(0.07)	(0.34)	(0.81)	(0.16)	(0.30)	(0.50)
R	0.09	0.15	0.11	−0.40	0.28	0.07
	(0.42)	(0.24)	(0.43)	(0.04)	(0.28)	(0.29)
HSR	0.17	0.07	0.04	−0.12	−0.11	0.04
	(0.14)	(0.57)	(0.79)	(0.53)	(0.68)	(0.49)
S	0.17	0.20	0.19	0.24	−0.13	0.13
	(0.13)	(0.11)	(0.17)	(0.23)	(0.63)	(0.04)
HIR	0.19	0.14	0.08	0.00	−0.13	0.08
	(0.09)	(0.25)	(0.56)	(1.00)	(0.63)	(0.22)

Legend: TD—total distance; LIR—low-intensity running; R—running; HSR—high speed running; S—sprinting, HIR—high-intensity running; CD—central defenders, FB—full backs, CM—central midfielders, WM—wide midfielders, FW—forwards.

## Data Availability

Data will be provided to all interested parties upon reasonable request.
